# Clinical outcomes of femoral neck fractures in nongeriatric patients: a comparative analysis of parallel screws, alpha fixation and femoral neck system

**DOI:** 10.1007/s00264-025-06671-5

**Published:** 2025-10-21

**Authors:** Dajun Jiang, Jiaqing Cao, Jinhui Zhao, Yuquan Bian, Shizan He, Weitao Jia

**Affiliations:** 1 Shanghai Sixth People’s Hospital Affiliated to Shanghai Jiao Tong University School of Medicine, Shanghai, China; 2 Shanghai Tenth People’s Hospital, School of Medicine, Tongji University, Shanghai, China

**Keywords:** Femoral neck fracture, Nongeriatric, Alpha fixation, Femoral neck system, Fixation failure

## Abstract

**Purpose:**

Femoral neck fractures in nongeriatric patients pose a significant clinical challenge due to the high clinical failure rate. To address this, Alpha fixation and the Femoral Neck System (FNS) were developed but seldom been compared head-to-head. The purpose of this study was to compare the clinical prognosis of these two methods with traditional parallel screws.

**Methods:**

This retrospective cohort study included 341 patients aged 18–65 years, treated between June 2020 and June 2023. Patients were grouped by fixation strategies: (1) parallel screws (*n* = 206), (2) Alpha fixation (*n* = 73), and (3) FNS (*n* = 62). Fixation failure (nonunion, severe femoral neck shortening, varus collapse) was compared as primary clinical outcome using univariate and multivariate analyses. Secondary outcomes included avascular necrosis and reoperation rates. Analyses were stratified by Pauwels classification.

**Results:**

Fixation failure rates were highest with parallel screws (21.4%), intermediate with FNS (12.9%), and lowest with Alpha fixation (9.6%). Multivariate analysis showed significantly lower fixation failure with Alpha fixation compared to parallel screws (adjusted OR = 0.29, 95% CI: 0.10–0.73, *p* = 0.014). Alpha fixation significantly reduced femoral neck shortening (*p* = 0.017), whereas FNS significantly reduced varus collapse (*p* = 0.013). In Pauwels type III fractures, Alpha fixation and FNS both significantly reduced fixation failure rates compared to parallel screws; no difference was found in Pauwels types I–II.

**Conclusions:**

Alpha fixation and FNS significantly outperformed parallel screws in reducing fixation failure in vertical femoral neck fractures among nongeriatric patients. Alpha fixation showed advantages in limiting femoral neck shortening, whereas FNS more effectively prevented varus collapse. For stable fractures, conventional parallel screws remain a reasonable choice.

## Introduction

Femoral neck fractures in non-geriatric patients constitute 3% to 10% of such injuries [1, 2]. These fractures are clinically challenging due to their high-energy traumatic nature, unfavourable biomechanical environment, tenuous vasculature [3], and severe fracture types [4]. Young patients often have demanding functional requirements for work and recreational activities. Despite treatment efforts, clinical prognosis remains unsatisfactory, with reported clinical failure rates of 37% [5], non-union rates of 21.1% [6], avascular necrosis rates of 14.3% [7], and significant decreases in functional scores [8]. Alarmingly, 32.1% of these patients undergo major reconstructive surgery, with 20.3% eventually requiring hip arthroplasty [9]. The consequences of these complications are catastrophic, severely affecting hip function and quality of life, and imposing a significant psychological and emotional burden on patients, their families, and society.

Internal fixation strategies for femoral neck fractures can be broadly categorized into two types: cannulated screw and fixed angle devices (such as sliding hip screws). Three parallel screws, which function as load-sharing implants, offer advantages such as minimal invasiveness and dynamic compression [10, 11]. However, it is insufficient to confront shear force in more complex fracture types. Fixed angle devices, on the other hand, act as load-bearing implants, providing greater stiffness [12] and anti-varus capability [13] but are associated with more surgical violence and an increased incidence of avascular necrosis (AVN) [14]. To improve clinical outcomes for these patients, it is crucial to achieve both minimal invasiveness and mechanical stability. In pursuit of this goal, orthopaedic surgeons have continuously optimized fixation methods.

Our study team had conducted extensive clinical studies on this issue, leading to the pioneering introduction of the Alpha fixation method [15, 16]. This novel approach has demonstrated superior interfragmentary stability [10, 16] and a reduced fixation failure rate [10, 15] compared to conventional fixation methods. Concurrently, the Femoral Neck System (FNS) has emerged and gained widespread adoption, supported by biomechanical [12] and clinical evidence [17] suggesting its enhanced stability and improved outcomes over traditional three-screw fixation. Despite these advancements, there is a paucity of head-to-head comparative studies between Alpha fixation and FNS.

The aim of this study was to compare the clinical prognosis of femoral neck fractures treated with three different strategies: FNS, alpha fixation, and parallel screws. Additionally, we seek to analyze the differences in prognosis among these internal fixation methods, which possess varying mechanical properties, across different types of fractures.

## Patients & methods

### Participants

This retrospective cohort study included patients from a national orthopaedic clinical centre between June 2020 and June 2023. The eligibility criteria were: (1) age between 18 and 65 years; (2) treatment with three parallel screws, Alpha fixation or FNS; (3) complete imaging data. Patients were excluded if they had (1) pathological or concomitant proximal femoral fractures or (2) received less than two years of follow-up.

### Study group

A total of 341 patients were included in the final analysis. These patients were categorized into three internal fixation groups: three parallel screws (*n* = 206), Alpha fixation (*n* = 73), and FNS (*n* = 62). In order to ensure the satisfactory surgical quality, all surgeries were performed by the same group of senior orthopaedic surgeons. Closed reduction was attempted first. Reduction quality was evaluated based on Garden index. For patients with unacceptable reductions, an open reduction procedure was performed using a modified Smith-Peterson approach. For patients in the parallel screw group, three partially threaded cannulated screws were typically used and implanted in an inverted triangle configuration. For patients in the Alpha group [15], a single cross screw was added to the conventional three parallel screws, yielding on anteroposterior radiographs a construct resembling the Greek letter alpha (α). The cross screw was inserted first, beginning 2–5 mm proximal to the vastus ridge, directed toward the inferior portion of the femoral head, and oriented perpendicular to the fracture line. For patients in the FNS group [18], the FNS power rod was inserted as close as possible to the central axis of the femoral neck [18]. The lateral plate was then attached to the femoral shaft and fixed with one or two distal locking screws, depending on bone quality and fracture configuration. An anti-rotation screw was implanted when additional rotational stability was required.

Postoperatively, patients were advised against any weight-bearing activities for the first three months. Gradual partial weight-bearing was permitted only if radiographic evaluations confirmed acceptable bone union. Follow-up assessments were scheduled at six weeks, three months, and one year postoperatively, and annually thereafter. At each follow-up, AP and lateral radiographs were routinely performed.

### Variables

Demographic information was collected, including age, sex, smoking status (defined as individuals who had smoked 100 or more cigarettes in their lifetime and who smoked at least one cigarette per day for a minimum of 6 months) [19], BMI, and comorbidities such as diabetes mellitus and hypertension. Fracture severity was assessed using two classification systems: the Garden classification for displacement degree (**nondisplaced**: Garden Types I and II; **displaced**: Garden Types III and IV) and the Pauwels classification [20] for fracture stability (**stable**: Pauwels Types I and II; **vertical**: Pauwels Type III). reduction quality (good or poor). Reduction quality was classified as good or poor; poor reduction (borderline acceptable or unacceptable) was defined as an alignment angle either < 155° or >180°.

### Clinical outcomes

In the current study, the primary outcome was fixation failure including non-union, femoral neck shortening, varus collapse. Non-union was defined as fracture redisplacement or the absence of radiographic evidence of union at six months postoperatively. Femoral neck shortening and varus collapse were defined as a decrease of over 10 mm in femoral neck length [21] or a reduction of 10° in the femoral neck shaft angle [22], as measured on anteroposterior radiographs.

Additionally, late complications such as avascular necrosis (defined as Type 2b or greater according to the Ficat classification [23]) were recorded. Furthermore, reoperations were documented, including secondary arthroplasty, revision surgeries (such as free vascular fibular graft and re-osteosynthesis), and implant removal.

### Statistical analysis

All statistical analyses were performed using R software (version 4.3.0). First, univariate analysis was conducted to examine the differences in baseline data distribution and clinical outcomes among the three fixation groups. Following this, multivariate analysis was conducted to investigate the significant differences between various fixation methods and clinical outcomes. Variables with a *p*-value < 0.1 in the baseline information and those closely related to prognosis were included as confounding factors. Additionally, subgroup analyses were performed based on variables demonstrating significant baseline differences, with statistical significance defined as *p* < 0.05 (two-tailed tests).

## Results

A total of 341 patients participated in this study, with 206 patients receiving parallel screw fixation, 73 patients undergoing Alpha fixation, and 62 patients treated with the FNS. The median age of the included patients was 53 years (interquartile range, 45–57). Baseline demographic and fracture characteristics for each group are presented in Table 1. Statistically significant differences were observed among the three groups regarding fracture stability (*p* = 0.001).

**Table 1:** Baseline characters of participated patients

		Parallel screws fixation	Alpha fixation	Femoral neck system	*P*-Value
Patients		206	73	62	
Age		54.0 (48.0–57.0)	51.0 (42.0–55.0)	53.0 (45.2–59.0)	0.087
Sex					
	Female	107 (51.9%)	28 (38.4%)	26 (41.9%)	0.089
	Male	99 (48.1%)	45 (61.6%)	36 (58.1%)	
BMI		23.4 (20.8–25.7)	22.8 (21.3–24.9)	22.7 (20.1–27.0)	0.811
Fracture displacement					
	Nondisplaced	79 (38.3%)	19 (26.0%)	26 (41.9%)	0.103
	Displaced	127 (61.7%)	54 (74.0%)	36 (58.1%)	
Fracture stability					
	Stable	98 (47.6%)	17 (23.3%)	25 (40.3%)	0.001
	Unstable	108 (52.4%)	56 (76.7%)	37 (59.7%)	
Smoker					
	No	146 (70.9%)	47 (64.4%)	39 (62.9%)	0.375
	Yes	60 (29.1%)	26 (35.6%)	23 (37.1%)	
Hypertension					
	No	166 (80.6%)	59 (80.8%)	45 (72.6%)	0.367
	Yes	40 (19.4%)	14 (19.2%)	17 (27.4%)	
Diabetes mellitus					
	No	179 (86.9%)	66 (90.4%)	55 (88.7%)	0.716
	Yes	27 (13.1%)	7 (9.6%)	7 (11.3%)	
Reduction quality					
	Good	180 (87.4%)	66 (90.4%)	57 (91.9%)	0.542
	Poor	26 (12.6%)	7 (9.6%)	5 (8.1%)	

**Fig. 1:**
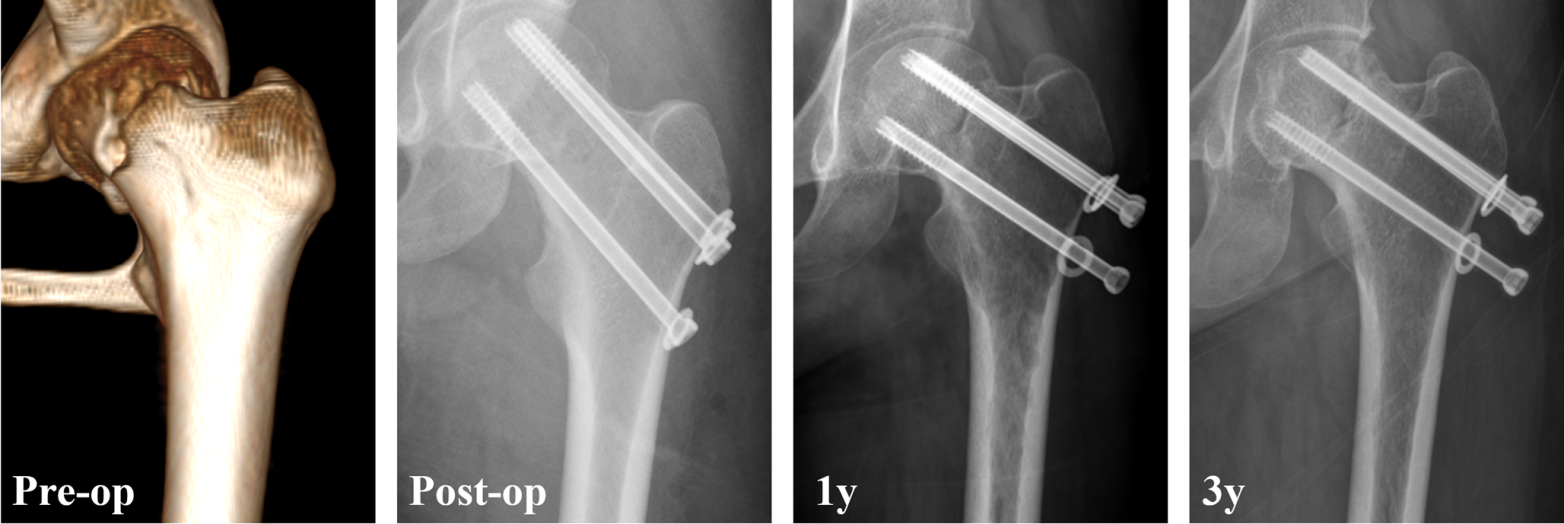
A 32-year-old female who sustained a Pauwels III femoral neck fracture was treated with three cannulated screws. The radiograph taken 1 year postoperatively revealed fixation failure, including femoral neck shortening and non-union. Follow-up at 3 years demonstrated the development of avascular necrosis

**Fig. 2:**
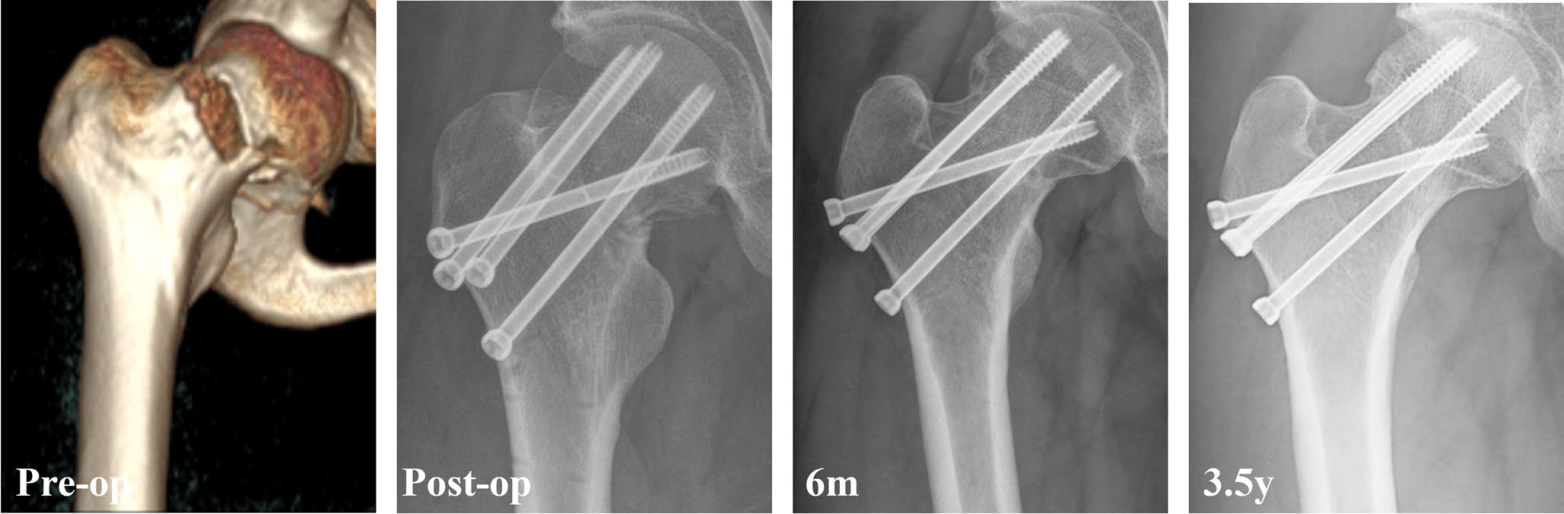
A 44-year-old male who sustained a Pauwels III femoral neck fracture was treated with alpha fixation. The radiograph taken 6 months and 2 years postoperatively revealed fracture union without complications

**Fig. 3:**
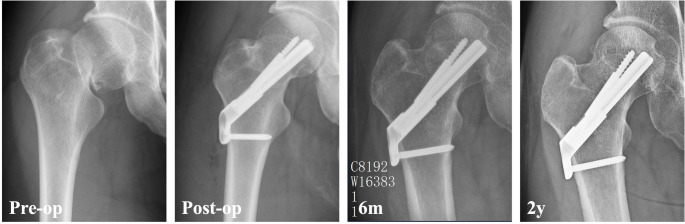
A 33-year-old male who sustained a Pauwels III femoral neck fracture was treated with femoral neck system. The radiograph taken 6 months and 2 years postoperatively revealed fracture union without complications

During follow-up, 17.3% of patients experienced fixation failure, including 10.6% with non-union, 12.9% with severe femoral neck shortening and 8.5% with varus collapse (Table 2). Additionally, 17.6% of patients developed avascular necrosis, and 19.4% underwent reoperation. Univariate analysis revealed that the fixation failure rate was highest in the parallel screws group (21.4%), followed by the FNS group (12.9%), and was lowest in the Alpha fixation group (9.6%) (Figs. 1, 2 and 3). Significant differences were observed between the Alpha fixation and parallel screw groups regarding fixation failure (*p* = 0.033) and femoral neck shortening (*p* = 0.017). Additionally, the FNS demonstrated a significantly lower rate of varus collapse compared with parallel screws (*p* = 0.013). After adjusting for confounding variables, multivariate analysis (Table 3) demonstrated that alpha fixation (adjusted OR = 0.29, 95% CI: 0.10–0.73; *p* = 0.014) was significantly associated with a lower rate of fixation failure, whereas FNS was not (*p* = 0.227).

**Table 2:** Univariate differences between clinical prognosis and different internal fixation strategies

	Total	G1: Parallel screws	G2: Alpha fixation	G3: Femoral neck system	*P*-Value	G1 vs. G2	G1 vs. G3	G2 vs. G3
Num	341	206	73	62				
Fixation failure	59 (17.3%)	44 (21.4%)	7 (9.6%)	8 (12.9%)	0.441	0.033	0.199	0.591
Non-union	36 (10.6%)	26 (12.6%)	5 (6.8%)	5 (8.1%)	0.301	0.201	0.375	1.000
Femoral neck shortening	44 (12.9%)	34 (16.5%)	4 (5.5%)	6 (9.7%)	0.038	0.017	0.226	0.512
Varus collapse	29 (8.5%)	24 (11.7%)	4 (5.5%)	1 (1.6%)	0.027	0.174	0.013	0.374
Avascular necrosis	60 (17.6%)	36 (17.5%)	14 (19.2%)	10 (16.1%)	0.896	0.726	1.000	0.822
Reoperation	66 (19.4%)	42 (20.4%)	14 (19.2%)	10 (16.1%)	0.757	0.867	0.583	0.822

**Table 3:** Multivariable analysis between primary outcome and fixation strategies

	Crude-OR	Adjusted-OR^*^	*p*
Parallel screws	1	1	1
Alpha fixation	0.33 (0.12–0.76)	0.29 (0.10–0.73)	0.014
Femoral neck system	0.55 (0.23–1.18)	0.55 (0.2–1.39)	0.227

Confounding variables included age, fracture stability, sex, fracture displacement, and reduction quality

Subsequently, a subgroup analysis of the entire cohort was performed based on fracture stability (Table 4). For vertical femoral neck fractures, the overall fixation failure rate was high at 24.3%. Significant differences were observed among the three groups in terms of fixation failure (*p* = 0.002), severe femoral neck shortening (*p* = 0.009), and varus collapse (*p* = 0.023). Specifically, compared to parallel screws, both Alpha fixation and the FNS had significantly lower rates of fixation failure (Alpha: 10.7%; FNS: 18.9%; parallel screws: 33.3%) and femoral neck shortening (Alpha: 7.1%; FNS: 16.2%; parallel screws: 26.9%), with *p*-values < 0.05. Additionally, the FNS had a significantly lower rate of varus collapse (2.7% vs. 17.6%, *p* = 0.023) compared to parallel screws. However, there was no significant difference in varus collapse rates between Alpha fixation and parallel screws (*p* = 0.112). When comparing Alpha fixation directly with FNS, no significant differences were observed in complication rates. However, Alpha fixation tended to have lower rates of fixation failure (10.7% vs. 18.9%, *p* > 0.05), nonunion (7.1% vs. 10.8%, *p* > 0.05), and femoral neck shortening (7.1% vs. 16.2%, *p* > 0.05), but showed slightly higher varus collapse rate (7.1% vs. 2.7%, *p* > 0.05). No significant differences (*p* > 0.05) were found among the three fixation methods in terms of avascular necrosis and reoperation rates.

**Table 4:** Clinical outcomes among three fixation strategies in vertical femoral neck fractures

		Total	Parallel screws	Alpha fixation	Femoral neck system	*P*-Value
Num		201	108	56	37	
Fixation failure		49 (24.3%)	36 (33.3%)	6 (10.7%)	7 (18.9%)	0.002
	Non-union	28 (13.9%)	20 (18.5%)	4 (7.1%)	4 (10.8%)	0.114
	Femoral neck shortening	39 (19.4%)	29 (26.9%)	4 (7.1%)	6 (16.2%)	0.009
	Varus collapse	24 (11.9%)	19 (17.6%)	4 (7.1%)	1 (2.7%)	0.023
Avascular necrosis		51 (25.4%)	29 (26.9%)	13 (23.2%)	9 (24.3%)	0.868
Reoperation		56 (27.9%)	34 (31.5%)	13 (23.2%)	9 (24.3%)	0.464

For stable (Pauwels I-II) femoral neck fractures, the overall fixation failure rate was relatively low (7.2%), and there were no significant (*p* > 0.05) differences in complication or reoperation rates among the three groups.

## Discussion

In this study, we investigated clinical outcomes of 341 patients treated with parallel screws, Alpha fixation, or the FNS. The data suggested that FNS and Alpha are reliable alternative to parallel screws in the treatment of femoral neck fractures. For vertical fracture type, both of FNS and Alpha fixation can significantly reduce fixation failure rate, especially in terms of femoral neck shortening and varus collapse. However, it is noteworthy that compared to FNS, Alpha fixation exhibited a trend toward lower rates of nonunion (7.1% vs. 10.8%), femoral neck shortening (7.1% vs. 16.2%), and fixation failure (10.7% vs. 18.9%), but a slightly higher rate of varus collapse (7.1% vs. 2.7%). For stable fracture types, the fixation failure rate was relatively low and there were no significant differences among the three fixation groups.

In nongeriatric patients with femoral neck fractures, the high-energy traumatic nature of the injury results in significant shearing and varus stresses at the fracture site. While the traditional use of three screws is easy handling, it often fails to counteract shear stress effectively [24], leading to bone cutting and insufficient mechanical stability for complex fractures. This issue is particularly prominent in vertical femoral neck fractures, resulting in a 24.3% fixation failure rate, which includes 13.9% non-union, 19.4% femoral neck shortening, and a 11.9% varus collapse rate. These results are far from satisfactory and consistent with those observed in previous studies [15, 25].

To address these challenges, our study team first proposed the Alpha fixation technique and pioneered the first cohort study [15], which demonstrated enhanced clinical prognosis. Our in-depth analysis [10, 16] of the mechanical principles revealed that, compared to traditional three-screw fixation, Alpha fixation optimizes load transmission and enhances resistance to shear stress. Additionally, this technique can prevent the “sliding effect”, increase screw-anchoring force, enhance cortical support, and ultimately improves ***interfragmentary stability*** [10, 16]. According to our previous analysis [10, 13], FNS, as a load-bearing fixed-angle device, has the advantage of ***construct stability***. Biomechanical studies [12, 26] have also confirmed that FNS provides greater axial, bending, and torsional stiffness.

In the current study, both Alpha fixation and FNS reduced fixation failure rates, highlighting their applicability in treating vertical femoral neck fractures. Compared to FNS, Alpha fixation demonstrated numerically lower rates of femoral neck shortening and nonunion, although the differences did not reach statistical significance. This suggests that Alpha fixation’s superior interfragmentary stability, as previously reported [13], may play a crucial role in maintaining local mechanical environment necessary for fracture healing. However, we observed that FNS exhibited a significantly lower varus collapse rate, reflecting its strength as a fixed-angle device with enhanced construct stability [13]. This characteristic might be particularly beneficial in managing comminuted fractures that are prone to high varus deformation forces. Therefore, for unstable femoral neck fractures, surgeons should further explore the specific biomechanical properties and indications of various fixation implants to select the optimal fixation strategy. Additionally, FNS still requires improvements for treating vertical femoral neck fractures, such as using double screws [27] for distal locking or adding an anti-rotation screw [28], to enhance fixation stability and improve clinical outcomes.

For stable fractures, there is no significant difference among the three fixation methods, and the outcomes are generally satisfactory. FNS has the advantage of shorter surgical time but comes at a higher cost compared to the three-screw fixation. Alpha fixation does not show a significant advantage in managing these fractures and has a slightly higher fixation failure rate compared to FNS. Therefore, for these types of fractures, surgeons should prioritize their familiarity with the fixation method, surgical time, and cost-effectiveness when selecting a treatment approach.

Despite decades of development in novel internal fixation devices for femoral neck fractures, no significant differences have been observed in the long-term complications, particularly AVN, among various fixation strategies. Most of the recent fixation strategies, such as alpha fixation and FNS, primarily address mechanical issues and enhance the stability of the fracture site. However, they seldom tackle the biological aspects. Previous research [15] has indicated that the occurrence of AVN is mainly dependent on the extent of blood supply disruption at the time of fracture. Therefore, future advancements in the treatment of femoral neck fractures are likely to focus more on biological issues, such as the restoration of proximal femoral blood supply.

This study has the following limitations. First, the current study primarily focuses on the mechanical complications associated with different fixation methods, with only those with at least two years of follow-up included in the analysis. Consequently, long-term outcomes such as AVN and reoperation rates may not be accurately reflected in this cohort. Additionally, we did not assess the quality of fixation, such as the positioning of the implants. Some cases had suboptimal positioning, with the three-screw fixation scoring less than 5 on the IMPO scale [29] or the FNS not being placed in the center/inferior-center position [30], which could influence the results. However, all procedures were performed by surgeons of the same rank, helping to reduce potential confounding bias. Furthermore, since FNS was officially introduced in our medical center only in recent years, the sample size for this method is relatively small, potentially resulting in insufficient statistical power for some comparisons.

Femoral neck fractures in nongeriatric patients remain a challenging clinical issue, with an overall fixation failure rate of 17.3%. In this study, both Alpha fixation and the FNS demonstrated significantly lower fixation failure rates compared to parallel screws in vertically oriented femoral neck fractures. However, for stable fractures, no significant differences were observed among the three fixation methods, indicating that conventional parallel screw fixation may remain sufficient for these patients. In terms of specific complications, Alpha fixation exhibited a slight advantage over FNS in minimizing femoral neck shortening, whereas FNS was superior in reducing the risk of varus collapse. Further research is needed to refine the surgical techniques for FNS to enhance stability and broaden its indications. Prospective large-scale clinical studies should identify the most suitable fixation strategy for different fracture types and populations, ultimately improving outcomes for nongeriatric patients and reducing societal burden.

## Data Availability

No datasets were generated or analysed during the current study.
